# Horse-racing injuries in children before and after the introduction of safety regulations in Mongolia

**DOI:** 10.5365/wpsar.2025.16.4.1195

**Published:** 2025-11-24

**Authors:** Gerelmaa Gunsmaa, Uugantsetseg Gurbazar, Tumen Ulzii Badarch, Masao Ichikawa

**Affiliations:** a Department of Global Public Health, Institute of Medicine, University of Tsukuba, Tsukuba, Ibaraki, Japan.; b Department of Public Health and Traditional Medicine, Darkhan-Uul Medical School, Mongolian National University of Medical Sciences, Darkhan, Mongolia.; c Department of Statistics and Surveillance, National Trauma and Orthopedic Research Center, Ulaanbaatar, Mongolia.

## Abstract

The aim of this study was to assess the effectiveness of safety regulations governing traditional Mongolian horse racing on the frequency and severity of injuries among child jockeys. Regulations introduced in 2019 mandate the wearing of helmets and protective clothing, prohibit the participation of jockeys aged < 7 years, and ban horse racing during the cold season (November–April). National injury surveillance data were used to compare the profile of injuries that occurred among children aged < 15 years in the 4-year periods before and after the introduction of the regulations (2015–2018 and 2019–2022) and to investigate whether injuries continued to occur among underage children and during the banned season. The proportion of head injuries among injured children was calculated before and after the regulations were introduced. During the study periods, 6309 animal-riding injuries were recorded among children aged 3–14 years; 2539 occurred before the regulations were introduced and 3770 occurred after. Following the introduction of the regulations, the proportion of injured children aged < 7 years decreased slightly. However, during 2019–2022, 294 animal-riding injuries were observed among underage children and 855 during the banned season. The proportion of head injuries among children with animal-riding injuries remained unchanged before and after the regulations were implemented (33.7% and 34.6%, respectively). The regulations have been ineffective. To reduce the burden of injuries among child jockeys, safety regulations need to be enforced throughout the year, and more stringent penalties for noncompliance should be imposed.

Horse racing is deeply rooted in Mongolian culture. (*1*, *2*) It has been an integral part of the celebration of key cultural events since the era of the Hun Dynasty, (*3*) during which time winning horses were revered as treasures. (*4*) Horse racing remains a beloved sport among Mongolians, and races take place across the country throughout the year. It is one of three traditional sports (alongside wrestling and archery) that form part of Naadam, (*5*) the country’s biggest and most important national festival, which is celebrated in July and is included on the Representative List of the Intangible Cultural Heritage of Humanity of the United Nations Educational, Scientific and Cultural Organization (UNESCO). (*6*)

Mongolian horse racing has three distinctive characteristics: long distances, natural terrain courses and child jockeys (**Fig. 1**). Race distances typically range from 10 km to 26 km, depending on the age of the horse. (*7*) For the child jockeys, who may be as young as 5 or 6 years, riding for such long distances over natural terrain is a high-risk activity. Moreover, child jockeys need only to have completed 1 month of training, and many participate in multiple races in a single day. Reportedly, in the first half of 2022, 835 horse races took place involving 33 818 child jockeys; 298 were injured, six seriously, and six lost their lives. (*8*)

**Fig. 1:**
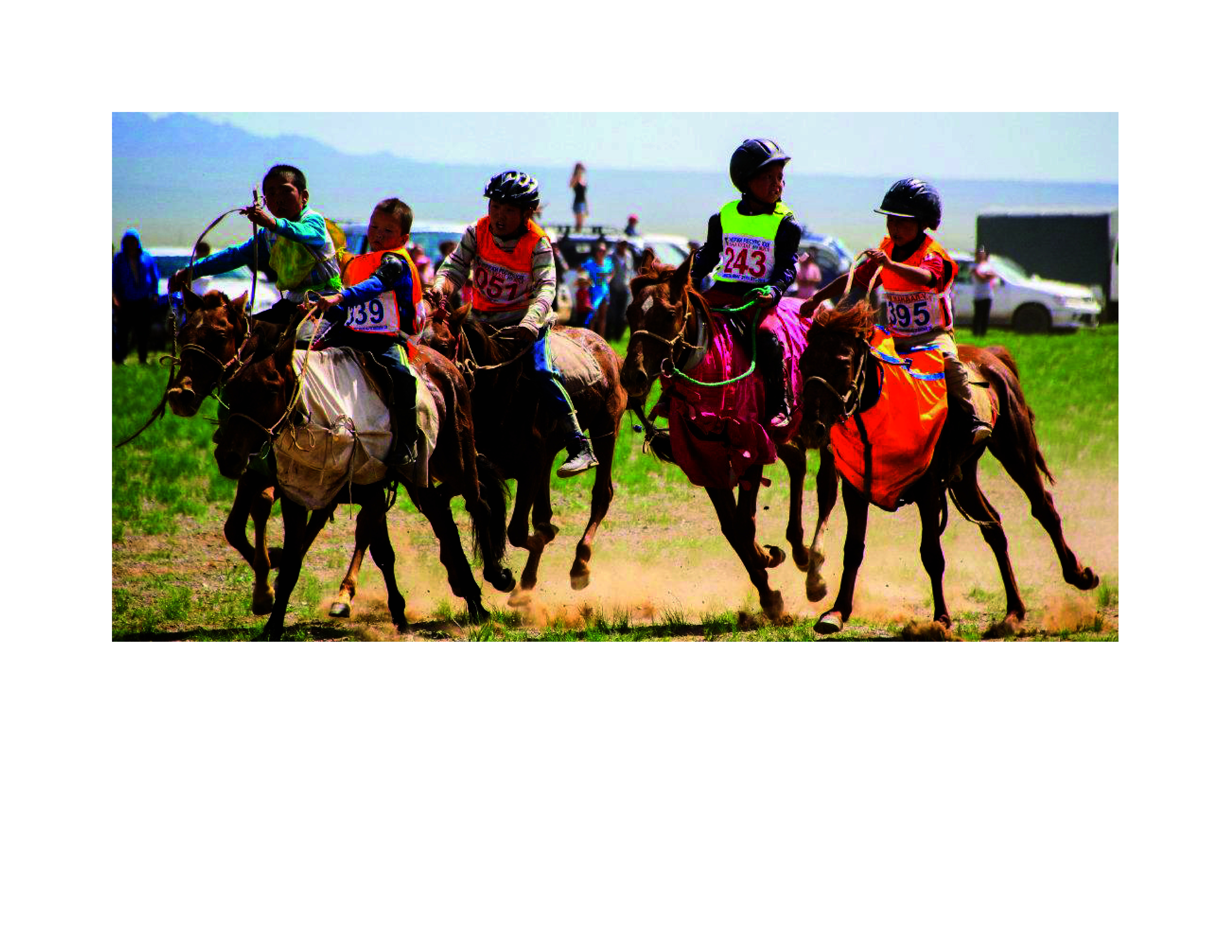
Mongolian child jockeys

[**insert** Figure 1]

To reduce the risk of injury among child jockeys, the Government of Mongolia introduced safety regulations in January 2019, mandating that all jockeys wear helmets along with gloves, boots and other protective gear during both races and training sessions. (*9*) The Government also prohibited children aged < 7 years from participating in races and banned horse racing during the cold season (November–April). (*7*, *10*)

In this study, national injury surveillance data were used to examine whether these regulations have achieved their goals and whether the requirement to wear a helmet has led to a reduction in head injuries among child jockeys.

## Methods

### Study setting

Mongolia has a population of approximately 3.4 million individuals, 32% of whom are children aged < 15 years. (*11*) In 2022, more than 190 000 households engaged in herding activities, managing 71 million livestock, including 4.8 million horses. (*12*) These animals play a pivotal role in the daily lives and livelihoods of nomads, providing transportation, food, clothing and other necessities.

### Data

Data about injuries from riding animals among children aged < 15 years were obtained for the period 2015–2022 from the database for National Surveillance on Injury, Mortality and Morbidity, which is maintained by the National Trauma and Orthopaedic Research Center. The national injury surveillance system routinely collects data from the medical records of all injured patients attending any of the more than 600 health facilities across the country, including 17 specialized hospitals, 5 regional hospitals, 22 provincial hospitals, 14 municipal district hospitals, 319 district hospitals and 224 family health centres, as well as private hospitals that provide trauma care. These data include information about patient demographics, injury severity and treatment outcomes. All health facilities must submit electronic records monthly. Injuries are coded according to the 10th revision of the World Health Organization’s International Classification of Diseases and Related Health Problems (ICD-10) by the Department of Statistics and Surveillance at the National Trauma and Orthopaedic Research Center.

For the purposes of this study, information was extracted from the surveillance database about the sex and age of patients, the place and date of injury, the affected body parts (e.g. head) and injury severity (i.e. fatal or non-fatal) for children whose injuries were coded as V80.0 (rider or occupant injured by fall from or being thrown from an animal or animal-drawn vehicle in a noncollision accident), V80.1 (rider or occupant injured in a collision with a pedestrian or animal), V80.7 (rider or occupant injured in a collision with other nonmotor vehicle, which includes an animal being ridden), V80.8 (rider or occupant injured in a collision with a fixed or stationary object) or V80.9 (rider or occupant injured in other and unspecified transport accidents, which include unspecified animal-rider accidents). We excluded V80.2 (those injured in a collision with a pedal cycle), V80.3 (injured in collision with a two- or three-wheeled motor vehicle), V80.4 (collision with a car, pick-up truck, van, heavy transport vehicle or bus), V80.5 (collision with any other specified motor vehicle) and V80.6 (collision with a railway train or railway vehicle) because they are unlikely to be related to horse racing. Head injuries were defined as “injuries to the head,” coded as S01–S09 to capture moderate to severe injuries. Head injuries coded as S00 (superficial injury of head) were excluded to ensure only the more severe cases were captured.

### Data analysis

The characteristics of children who sustained animal-riding injuries during the 4-year period before the introduction of the safety regulations (January 2015–December 2018) were compared with those during the 4-year period after (January 2019–December 2022) in terms of sex (male vs female), age group (< 7, 7–9, 10–12, 13–14 years), month of injury (November–April vs May–October), injury severity (fatal vs non-fatal) and type of injury (ICD-10 classification). To assess whether head injuries decreased after the introduction of the regulations in 2019, the proportion of head injuries among injured children was calculated, overall and by sex and age group, for the same two periods. The analysis was stratified by place of injury to determine whether there were differences in the frequency and type of injuries between urban and rural areas.

## Results

Between 2015 and 2022, 6309 injuries related to animal riding occurred among children aged 3–14 years. The majority (95.3%, 6014/6309) were classified as V80.0. Boys accounted for 91.1% (5748/6309) of all recorded injuries; 9.5% (598/6309) occurred in children aged < 7 years. Just over half of all injuries (55.5%, 3504/6309) occurred in rural areas, 21.0% (1328/6309) occurred during the banned season and 1.7% (109/6309) of injuries were fatal (**Table 1**).

**Table 1:** Injuries associated with riding an animal in children aged < 15 years before and after the introduction of horse racing safety regulations in 2019 by urban or rural area, Mongolia, 2015–2022

Characteristic	Urban				Rural				Total			
	Before (2015–2018)		After (2019–2022)		Before (2015–2018)		After (2019–2022)		Before (2015–2018)		After (2019–2022)	
**Total no.**	**1095**		**1710**		**1444**		**2060**		**2539**		**3770**	
**Sex**												
**Male**	**996**	**(91.0)**	**1568**	**(91.7)**	**1291**	**(89.4)**	**1893**	**(91.9)**	**2287**	**(90.1)**	**3461**	**(91.8)**
**Female**	**99**	**(9.0)**	**142**	**(8.3)**	**153**	**(10.6)**	**167**	**(8.1)**	**252**	**(9.9)**	**703**	**(9.2)**
**Age group (years)**												
** < 7 (banned age)**	**118**	**(10.8)**	**124**	**(7.3)**	**186**	**(12.9)**	**170**	**(8.3)**	**304**	**(11.9)**	**294**	**(7.8)**
**7–9**	**326**	**(29.8)**	**510**	**(29.8)**	**466**	**(32.3)**	**607**	**(29.5)**	**792**	**(31.2)**	**1117**	**(29.6)**
**10–12**	**368**	**(33.6)**	**695**	**(40.6)**	**488**	**(33.8)**	**809**	**(39.3)**	**856**	**(33.7)**	**1504**	**(39.9)**
**13–14**	**283**	**(25.8)**	**381**	**(22.3)**	**304**	**(21.1)**	**474**	**(23.0)**	**587**	**(23.1)**	**855**	**(22.7)**
**Month of injury**												
**November–April (banned season)**	**152**	**(13.9)**	**364**	**(21.3)**	**321**	**(22.2)**	**491**	**(23.8)**	**473**	**(18.6)**	**855**	**(22.7)**
**May–October**	**943**	**(86.1)**	**1346**	**(78.7)**	**1123**	**(77.8)**	**1569**	**(76.2)**	**2066**	**(81.4)**	**2915**	**(77.3)**
**Injury severity**												
**Fatal**	**4**	**(0.4)**	**5**	**(0.3)**	**45**	**(3.1)**	**55**	**(2.7)**	**49**	**(1.9)**	**59**	**(1.6)**
**Non-fatal**	**1091**	**(99.6)**	**1705**	**(99.7)**	**1399**	**(96.9)**	**2005**	**(97.3)**	**2490**	**(98.1)**	**3710**	**(98.4)**
**Injury classification (ICD-10 code)^a^**												
**V80.0**	**1094**	**(99.9)**	**1699**	**(99.4)**	**1319**	**(91.3)**	**1902**	**(92.3)**	**2413**	**(95.0)**	**3601**	**(95.5)**
**V80.1**	**1**	**(0.1)**	**0**	**(0.0)**	**23**	**(1.6)**	**42**	**(2.0)**	**24**	**(0.9)**	**42**	**(1.1)**
**V80.7**	**0**	**(0.0)**	**3**	**(0.2)**	**7**	**(0.5)**	**15**	**(0.7)**	**7**	**(0.3)**	**18**	**(0.5)**
**V80.8**	**0**	**(0.0)**	**8**	**(0.5)**	**35**	**(2.4)**	**49**	**(2.4)**	**35**	**(1.4)**	**57**	**(1.5)**
**V80.9**	**0**	**(0.0)**	**0**	**(0.0)**	**60**	**(4.2)**	**52**	**(2.5)**	**60**	**(2.4)**	**52**	**(1.4)**

ICD-10: International Statistical Classification of Diseases and Related Health Problems, 10th revision.

Values are *n*(%).

^a^ V80.0: injured by fall from or being thrown from animal or animal-drawn vehicle in noncollision accident; V80.1: injured in collision with pedestrian or animal; V80.7: injured in collision with other nonmotor vehicle, which includes an animal being ridden; V80.8: injured in collision with fixed or stationary object; V80.9: injured in other and unspecified transport accidents, which include unspecified animal-rider accidents.

A total of 2539 injuries occurred during the 4 years before the regulations were implemented, while 3770 occurred in the subsequent 4-year period. While the overall number of injuries increased, the proportion of injured children aged < 7 years was lower in the 4-year period following the introduction of regulations in both urban and rural areas (urban areas: 10.8% [118/1095] vs 7.3% [124/1710]; rural areas: 12.9% [186/1444] vs 8.3% [170/2060]). In contrast, the proportion of injuries that occurred during the banned months increased, most noticeably in urban areas (13.9% [152/1095] vs 21.3% [364/1710]). The distribution of injuries by sex, injury severity and ICD-10 classification remained largely unchanged across the two periods (**Table 1**).

The proportion of head injuries among all injuries was higher in rural areas than in urban areas. This proportion did not change markedly between the two study periods; in urban areas, head injuries accounted for 24.0% (259/1095) of injuries before and 25.0% (427/1710) after the regulations, and in rural areas for 41.0% (598/1444) before and 43.0% (876/2060) after (**Table 2**). In both periods, the proportion of head injuries was similar between boys and girls but was higher among younger children.

**Table 2:** Head injuries as a proportion of all injuries associated with riding an animal among children aged < 15 years before and after the introduction of horse racing safety regulations in 2019 by urban or rural area, Mongolia, 2015–2022

Characteristics	Before (2015–2018)					After (2019–2022)				
	All injuries		Head injuries		% head injuries	All injuries		Head injuries		% head injuries
**Urban areas**										
**Total no.**	**1095**	**(100.0)**	**259**	**(100.0)**	**24.0**	**1710**	**(100.0)**	**427**	**(100.0)**	**25.0**
**Sex**										
**Male**	**996**	**(91.0)**	**237**	**(92.0)**	**24.0**	**1568**	**(92.0)**	**395**	**(93.0)**	**25.0**
**Female**	**99**	**(9.0)**	**22**	**(8.0)**	**22.0**	**142**	**(8.0)**	**32**	**(7.0)**	**23.0**
**Age group (years)**										
** < 7 (banned age)**	**118**	**(10.0)**	**34**	**(13.0)**	**29.0**	**124**	**(7.0)**	**35**	**(8.2)**	**28.0**
**7–9**	**326**	**(30.0)**	**97**	**(37.0)**	**30.0**	**510**	**(30.0)**	**156**	**(37.0)**	**31.0**
**10–12**	**368**	**(34.0)**	**82**	**(32.0)**	**22.0**	**695**	**(41.0)**	**162**	**(38.0)**	**23.0**
**13–14**	**283**	**(26.0)**	**46**	**(18.0)**	**16.0**	**381**	**(22.0)**	**74**	**(17.0)**	**19.0**
**Month of injury**										
**November–April (banned season)**	**152**	**(14.0)**	**63**	**(24.0)**	**42.0**	**364**	**(21.0)**	**110**	**(26.0)**	**30.2**
**May–October**	**943**	**(86.0)**	**196**	**(76.0)**	**21.0**	**1346**	**(79.0)**	**317**	**(74.0)**	**24.0**
**Rural areas**										
**Total no.**	**1444**	**(100.0)**	**598**	**(100.0)**	**41.0**	**2060**	**(100.0)**	**876**	**(100.0)**	**43.0**
**Sex**										
**Male**	**1291**	**(89.0)**	**526**	**(88.0)**	**41.0**	**1893**	**(92.0)**	**803**	**(92.0)**	**42.0**
**Female**	**153**	**(11.0)**	**72**	**(12.0)**	**47.0**	**167**	**(8.0)**	**73**	**(8.0)**	**44.0**
**Age group (years)**										
** < 7 (banned age)**	**186**	**(13.0)**	**97**	**(16.0)**	**52.0**	**170**	**(8.0)**	**85**	**(10.0)**	**50.0**
**7–9**	**466**	**(32.0)**	**186**	**(31.0)**	**40.0**	**607**	**(29.0)**	**284**	**(32.0)**	**47.0**
**10–12**	**488**	**(34.0)**	**199**	**(33.0)**	**41.0**	**809**	**(39.0)**	**353**	**(40.0)**	**44.0**
**13–14**	**304**	**(21.0)**	**116**	**(20.0)**	**38.0**	**474**	**(23.0)**	**154**	**(18.0)**	**32.0**
**Month of injury**										
**November–April (banned season)**	**321**	**(22.0)**	**152**	**(25.0)**	**47.0**	**491**	**(24.0)**	**249**	**(28.0)**	**51.0**
**May–October**	**1123**	**(78.0)**	**446**	**(75.0)**	**40.0**	**1569**	**(76.0)**	**627**	**(72.0)**	**40.0**
**Total (urban and rural)**	**2539**	**(100.0)**	**857**	**(100.0)**	**33.7**	**3770**	**(100.0)**	**1303**	**(100.0)**	**34.6**

Values are *n*(%) unless otherwise indicated.

## Discussion

Despite the introduction of horse racing regulations banning the participation of child jockeys aged < 7 years and racing during the cold season, in the 4 years after introduction, 294 animal-riding injuries were recorded among underage children. Moreover, 855 children were injured during the banned season, up from 473 in the preceding 4-year period. Furthermore, safety regulations mandating helmet-wearing by child jockeys do not appear to have reduced either the number or the proportion of head injuries among children with animal-riding injuries. These findings are consistent between urban and rural areas but, notably, the number of children who sustained head injuries, especially young children, was much higher in rural areas than in urban areas.

The failure of helmet-wearing regulations to reduce head injuries may be due to a combination of factors. First, compliance with regulations about helmet-wearing is low. According to nationwide inspections conducted in 2020 and 2021, the proportion of 17 450 jockeys wearing helmets during races was 42% (General Agency for Specialised Inspection of Mongolia. Official letter No. 02/356, February 21, 2022. Ulaanbaatar, Mongolia. Not publicly available). A subsequent qualitative study reported that child jockeys often find helmets uncomfortable and so avoid wearing them. (*13*) Other possible reasons for low levels of compliance include a reluctance on the part of guardians and race organizers to abide by the regulations, possibly because of a lack of concern about safety and regulatory enforcement. Indeed, many horse races are unregistered, (*14*) and the fines for violating regulations are low, typically no more than 100 000 Mongolian tugrug (approximately US$ 30). (*15*) Second, although the new regulations stipulate that helmets must conform to Mongolian standards, we suspect that some child jockeys are wearing non-standard or loose-fitting helmets without chin straps. Considering the hard, rocky natural terrain of the race courses, such helmets are unlikely to afford sufficient protection in the event of a fall.

This study also provides evidence of noncompliance with the regulations about horse racing seasons and the age of child jockeys. Despite the ban on horse races during November–April, in 2022, horse races were held in eight of the country’s 21 provinces to celebrate the National Spring Festival (known as Tsagaan Sar) and the Lunar New Year, which in 2022 was at the end of February. (*16*, *17*) The increase in horse-related injuries after the introduction of the regulations might have resulted from an increasing number of such horse races due to commercialization of the races. Traditionally, horse racing was part of nomadic herders’ culture, with races held a few times a year during the summer festival, and children rode their own family’s horses. However, horse racing has now become a business and gambling may be involved. Horses are raised year-round under careful management, and children are employed as jockeys, with horses bred for racing commercially rather than in the traditional way.

Enforcement actions are clearly needed to increase compliance with safety regulations throughout the year, especially in rural areas where head injuries were more prevalent among injuries sustained while riding than they were in urban areas. Additionally, more stringent penalties for noncompliance should be imposed. Such policies have proven effective in other countries. For instance, in 2002, the Government of United Arab Emirates banned child jockeys aged < 15 years from participating in traditional camel races. Strict penalties were imposed on camel owners: a fine of 20 000 dirhams (approximately  US$ 5500) was levied for the first offence; a second offence incurred a 1-year suspension from racing; and third and subsequent offences resulted in imprisonment. (*18*) A single-centre study reported that there were no paediatric patients with injuries related to camel racing after the ban in United Arab Emirates, (*19*) although violations of the ban were reported in 2010. (*20*) Almost concurrent with the 2002 ban, robotic jockeys were developed to replace child jockeys, (*21*) and these have since become commonplace in camel races across United Arab Emirates. (*22*) We believe that switching to robotic jockeys would allow Mongolians to enjoy the ancient tradition of horse racing throughout the year, while protecting the safety and human rights of children.

This study has several limitations. First, ICD-10 codes, notably V80.0, are not specific to injuries incurred while participating in horse racing. However, our findings are not likely to be distorted because it is unlikely that the incidence of riding injuries unrelated to horse racing (e.g. in farming) changed substantially during the study period. Second, surveillance data did not capture injuries incurred during horse racing that were not treated at health-care facilities. Presumably, such injuries were mostly minor and did not require medical treatment. Conceivably, increased helmet-wearing may have reduced the severity of head injuries; had this been the case, we might have expected to see a drop in the proportion of head injuries among injured children after the introduction of regulations. However, we did not, most likely because compliance with helmet-wearing was low (General Agency for Specialised Inspection of Mongolia. Official letter No. 02/356, February 21, 2022. Ulaanbaatar, Mongolia. Not publicly available). Data on helmet use among injured children would have been helpful, as they would have allowed us to assess the relative contributions of not wearing a helmet and the inappropriate use of a helmet to the apparent ineffectiveness of the regulations in reducing head injuries. Finally, as we did not have information about the number of child jockeys who participated in the horse races, we were unable to estimate the incidence of injury per at-risk population during the study period. Nevertheless, we believe that the proportion of head injuries attributed to riding an animal as a percentage of all such injuries should be a sufficiently sound indicator of trends because the overall number of injuries could be considered a proxy for the at-risk population.

In conclusion, we consider the 2019 horse racing regulations to be ineffective. Head injuries did not decrease among children with animal-riding injuries following the introduction of the safety regulations; furthermore, riding injuries continued to occur among underage children and during the banned season. To reduce the burden of these injuries, year-round enforcement of safety regulations and more stringent penalties for noncompliance are recommended.
